# Supplementary material for: Sustained-input switches for transcription factors and microRNAs are central building blocks of eukaryotic gene circuits
Source: Genome Biol. 2013 Aug 23;14(8):R85. doi: 10.1186/gb-2013-14-8-r85 (PMC4054853; doi:10.1186/gb-2013-14-8-r85)
Supplement: Additional file 5 — HTML Browsable Motif Output. Zipped folder containing all WaRSwap and FANMOD motif output, viewable in a web browser. [file gb-2013-14-8-r85-S5.ZIP › HTML_browsable_motif_output/WaRSwap_ath_tair9/sigs_WSWRSLDraw_2-2000.pvals.heatmaps.html/motif_id_36_001001001_tftype_ath_upstream_-2000_0.html]

```
BG_MODEL = Rnew2
MOTIF_ID = 36_001001001
TF_TYPE = ath
UPSTREAM = -2000_0


PVals
FN_0.2	FN_0.4	FN_0.6	FN_0.8
dg_60.genes	0.383	0.462	0.99	0.004
dg_70.genes	0.391	0.443	0.993	0.004
dg_80.genes	0.408	0.44	0.995	0.002

ZScores
FN_0.2	FN_0.4	FN_0.6	FN_0.8
dg_60.genes	0.219	0.029	-2.161	2.27
dg_70.genes	0.206	0.088	-2.238	2.352
dg_80.genes	0.166	0.074	-2.256	2.289

StDevs
FN_0.2	FN_0.4	FN_0.6	FN_0.8
dg_60.genes	202.941	259.4	114.699	40.489
dg_70.genes	207.12	256.489	112.853	39.554
dg_80.genes	203.999	261.714	112.582	40.403
```
